# Factors Associated with COVID-19 Testing in Structurally Vulnerable Populations: An Exploratory Study in Southern Maine

**DOI:** 10.46804/2641-2225.1241

**Published:** 2025-12-18

**Authors:** Grace L. Price, Kathleen M. Fairfield, Sumayo Awale, Renee Fay-LeBlanc, Caroline Fernandes, Ann Marie R. Hess, Elizabeth A. Jacobs, Donna Lawlor, David Ngandu, Leslie Nicoll, Kinna Thakarar, Ellyn Touchette, Andrew Volkers, Gloria D. Sclar

**Affiliations:** a MaineHealth Institute for Research, Scarborough, Maine; b Department of Internal Medicine, Mainehealth Maine Medical Center, Portland, Maine; c Greater Portland Health, Portland, Maine; d Preble Street, Portland, Maine; e Department of Internal Medicine, University of California Riverside School of Medicine, Riverside, California; f Portland Community Free Clinic, Portland, Maine

**Keywords:** COVID-19, Health disparities, Public health, Access to health care

## Abstract

**Introduction:**

The COVID-19 pandemic disproportionately affected structurally vulnerable populations in the United States. COVID-19 testing was instrumental in controlling viral spread and linking people to treatment; however, testing rates were lower among racial and ethnic minority groups. Our objective was to identify factors associated with desired COVID-19 testing behavior among vulnerable populations.

**Methods:**

We conducted an exploratory cohort study of at-home COVID-19 testing between March 2022 and November 2023 in Portland, Maine. Partnering with trusted community organizations, we engaged participants from immigrant, housing-unstable, substance-using, and low-income/uninsured communities. Participants received 5 at-home COVID-19 tests every 8 weeks for 48 weeks. Participants completed a baseline survey and follow-up surveys every 4 weeks on COVID-19 exposures and symptoms, as well as use of COVID-19 tests, with additional questions every 8 weeks on behavioral factors (ie, risk perceptions, attitudes, norms) around COVID-19 testing. The primary outcome was “desired testing behavior score,” or the proportion of instances a participant tested when they should have based on guidance from the Centers for Disease Control and Prevention.

**Results:**

We enrolled 93 participants, of whom 39 (42%) were immigrants, 30 (32%) were unhoused or had a history of chronic homelessness, 28 (30%) reported substance use, and 60 (65%) were low income and/or uninsured. Overall, participants tested 66% of the recommended times. In bivariate regression, 4 factors (age, confidence in ability to use a COVID-19 test, perceived usefulness of testing, and commitment to testing) were significantly associated with desired testing behavior. However, these associations were not statistically significant in multivariable linear regression.

**Discussion and Conclusions:**

Participants from vulnerable communities engaged with COVID-19 testing when provided with at-home tests, but more research is needed to understand what factors drive testing behavior.

## 1. Introduction

In the United States, the COVID-19 pandemic has disproportionately affected vulnerable populations, including immigrant groups, racial and ethnic minority groups, rural dwellers,^1^ and people impacted by poverty, unstable housing, and substance use.^2^ Stark racial disparities emerged from the earliest days of the pandemic, with non-White individuals experiencing higher rates of COVID-19 infection,^3,4^ hospitalization,^5,6^ and death.^7^ Immigrant populations^8^ and people experiencing homelessness^9,10^ have also been disproportionately affected by the COVID-19 pandemic. The strain the pandemic placed on the health care system amplified serious health disparities that arise due to social determinants of health.^11^ The state of Maine had one of the highest rates of racial disparities in COVID-19 infections in the country through the first phase of the pandemic,^12^ and is an instructive site for piloting efforts to reduce these disparities.

COVID-19 testing is instrumental in controlling the spread of the virus and in linking people to treatment,^13^ although evidence supporting any specific testing method remains limited.^14^ Testing rates have generally been lower among racial and ethnic minority groups,^15–17^ and testing sites are frequently less accessible in lower-income areas.^18^ Therefore, programs to improve access to COVID-19 testing, particularly for vulnerable populations disproportionately impacted by the pandemic, are crucial to increasing testing and treatment. Access to testing sites has emerged as a consistent barrier to COVID-19 testing for vulnerable communities.^19–22^ Previous studies have examined efforts to increase testing by establishing testing clinics in low-income neighborhoods^23^ or providing support from community health workers for testing in vulnerable populations.^24^ During the pandemic, developing technology allowed a shift from exclusive use of laboratory-run tests accessed through testing sites to an increasing proportion of self-administered at-home tests. By early 2022, more than 20% of people in the United States with COVID-19 symptoms reported use of a home test.^25^ Various initiatives—through federal^26^ and state^27^ government, as well as health insurance providers,^28^ schools,^26^ and philanthropic organizations^29^—seek to distribute at-home COVID-19 tests widely. Thus, a clear understanding of how often people test when they have access to at-home tests, and what factors are associated with higher use of at-home tests, is needed.

To our knowledge, no studies to date have longitudinally engaged participants from vulnerable communities to assess COVID-19 testing behaviors and behavioral factors when participants are provided a consistent supply of at-home test kits and instructed on proper use. In this study, we aimed to: (1) describe the knowledge, attitudes, and behaviors toward COVID-19 testing over 1 year in underserved populations receiving at-home COVID-19 test kits; and (2) identify demographic characteristics and behavioral factors associated with desired COVID-19 testing behavior.

## 2. Methods

Using a community-based approach, we engaged participants from vulnerable communities in a cohort study focused on at-home COVID-19 testing. Participants received in-person instruction on testing, were provided with at-home COVID-19 test kits, and answered monthly surveys on testing use and behavioral factors. By following a community-engaged model, we aimed to build trust and understanding in order to effectively and ethically engage these vulnerable communities.^31,32^ We use the term “at-home testing” throughout because it is the predominant term in the literature. However, we acknowledge that many of our participants were housing-unstable and may have used self-administered tests in non-home settings.

### 2.1. Setting and participants

We conducted a longitudinal cohort study of at-home COVID-19 testing from April 2022 to November 2023, with recruitment focused on people in Portland, Maine. Recruitment occurred from April 13, 2022, to December 6, 2022. We aimed to engage a roughly equal number of participants from immigrant, housing-unstable, and low-income/uninsured communities. Individuals were eligible to participate if they were age 18 years and older and either: (1) an immigrant; (2) currently unhoused or had a history of chronic homelessness; or (3) received services through Portland Public Health (specifically the Portland Community Free Clinic, Sexually Transmitted Disease Clinic, or Needle Exchange).

### 2.2. Intervention

All participants attended an individual in-person enrollment session with research staff in which participants provided written informed consent, learned to perform a COVID-19 test, completed a baseline survey, and received 5 at-home COVID-19 tests. Participants continued to receive 5 at-home COVID-19 tests every 8 weeks for 48 weeks (30 tests total), regardless of whether they used all previous tests; this constituted the “testing program.” Approximately two-thirds of COVID tests were mailed to participants, whereas one-third were provided tests in person based on participant preferences. The MaineHealth Institutional Review Board approved the protocol in December 2021.

### 2.3. Measures

The baseline survey included study-specific questions about COVID-19 test use and behavioral factors (ie, risk perceptions, attitudes, norms). They also included the Rapid Acceleration of Diagnostics-Underserved Populations (RADx-UP) Common Data Elements, a set of standardized questions used in COVID-19 research funded by the National Institutes of Health (NIH) to build a repository of COVID-19 data (details of the Common Data Elements are described in Appendix A).^33^ Participants then completed a brief survey every 4 weeks on their COVID-19 exposures, symptoms, and use of COVID-19 tests. Every 8 weeks, participants completed a longer survey that included additional questions on behavioral factors around COVID-19 testing. The initial 8-week survey also included questions about substance-use behaviors that were adapted from the validated National Institute on Drug Abuse-Modified Alcohol, Smoking and Substance Involvement Screening (NIDA-ASSIST) survey.^34^ In total, each participant completed 1 baseline and 12 follow-up surveys over 11 months.

NIH translation services translated all recruitment and survey materials into 7 languages (Somali, Arabic, French, Kinyarwanda, Lingala, Portuguese, and Spanish) representing the top languages spoken by patients of Greater Portland Health (GPH), a federally-qualified health center that primarily serves the immigrant community). We securely hosted and stored the surveys on a REDCap database and distributed the REDCap survey link to participants over email, text, or WhatsApp (68% of completed surveys were distributed electronically). If requested, research staff also administered the surveys over the phone (14% of surveys) or in person (17% of surveys). Participants who did not respond to a survey were sent a reminder email/message up to 3 times over 6 days and called by research staff on day 7. Participants were considered lost to follow-up if they missed completing 3 consecutive surveys and could not be reached. Participants could receive up to $250 in grocery gift cards as compensation for their time participating in the study: a $50 gift card at enrollment, $20 gift cards every 8 weeks for completing surveys, and $40 gift cards for reaching the half-way point and end of the study. This compensation rate was based on typical practice by our institution and advice from the Community and Scientific Advisory Board and deemed appropriate by institutional review board review. Participants were informed that compensation was based solely on survey completion and not on survey responses or testing behavior.

The primary outcome was “desired testing behavior score.” In every survey, participants were asked whether, in the past month, they had a close contact exposure to a known case of COVID-19 or experienced symptoms of COVID-19 (reporting yes/no to a list of 12 symptoms), indicating an instance when testing was recommended by the Centers for Disease Control and Prevention (CDC).^13^ Following these questions, participants were asked if, in the past month, they had taken a COVID-19 test. The “desired testing behavior score” was then calculated as the proportion of surveys in which a participant reported taking a COVID-19 test when they reported an indication to test. For example, if a participant reported a symptom and/or exposure in 5 of their surveys but only reported testing in 3 of those surveys, then their desired testing behavior score was 3/5, or 0.6.

Independent variables examined in the analysis included vulnerability groups, behavioral factors, and demographic variables. The 4 dichotomous vulnerability groupings included: (1) immigrants, (2) unhoused or history of chronic homelessness, (3) low income and/or uninsured, and (4) substance use. The 5 behavioral factor scores related to COVID-19 testing and illness, measured using 5-point Likert scale questions, were: (1) confidence in ability to use tests, (2) perceived severity of illness from COVID-19, (3) perceived usefulness of testing, (4) norms around testing, and (5) commitment to testing. Behavioral factor questions were developed using input from prior qualitative work with this population^35^ and followed the Risks, Attitudes, Norms, Abilities, and Self-Regulation (RANAS) model of behavior change.^36^ The complete wording of the behavioral factor questions is presented in Appendix Table A2. Also, we analyzed 7 demographic characteristics: age, self-reported gender, race, employment status, education level, COVID-19 vaccination status, and self-reported health status.

### 2.4. Data analysis

We performed a descriptive analysis to characterize the demographics and vulnerability status of participants. We also stratified by vulnerability group to describe differences across groups. We examined whether COVID-19 testing behavioral factors differed by vulnerability group by using 2-sample t-tests between the group and non-group for each behavioral factor score. We used a significance of *P* less than .05 throughout.

We performed bivariate linear regressions to assess associations between desired testing behavior and each vulnerability, behavioral, and demographic factor. Factors significantly associated with desired testing behavior in the bivariate regressions were then included in a multivariable linear regression. The vulnerability score was also included, despite non-significance in bivariate analysis, because previous literature and earlier qualitative analysis in our community indicated these categories have a strong relationship with testing. A linear relationship was confirmed by a plot of residuals against the predicted values. There was no evidence of multicollinearity, as assessed by tolerance values greater than 0.1 and variance inflation factor values less than 10. The assumption of normality was met, as assessed by visual inspection of the Q-Q plot. Analyses were conducted using STATA version 17.0.

## 3. Results

We recruited a total of 120 adults. Of these adults, 18 did not complete enrollment, 3 declined to continue after initial enrollment, 3 were lost to follow-up, 2 could no longer continue due to medical issues, and 1 died, leaving 93 participants for our analysis. Participants completed 1034 of 1116 surveys (93% completion rate).

Table 1 presents the key demographic, health, and vulnerability characteristics of the study participants (for full demographic information, see Appendix Table A1). The mean participant age was 45.5 years, 54% were female, 45% were male, and 1% were nonbinary. Most participants identified as White and non-Hispanic (48%) or Black and African American (37%). Almost half of participants were employed, 39% reported having a bachelor’s degree or higher, 55% reported a household income below the federal poverty level, and 48% reported having public health insurance. Also, 50% reported excellent or very good health, and 90% had received at least 1 dose of a COVID-19 vaccine.

We categorized participants by vulnerability group. These groups were not mutually exclusive and were overlapping. Of the 93 overall participants, 39 (42%) were immigrants, 30 (32%) were unhoused or had a history of chronic homelessness, 28 (30%) reported substance use, and 60 (65%) were low income and/or uninsured. Most participants had more than 1 vulnerability, with 30 (32%) belonging to 2 vulnerability groups and 23 (25%) belonging to 3 or more groups. Conversely, 14 (15%) participants did not fall into any of these vulnerability groups.

Table 1 presents additional key demographics of the participants by vulnerability group. Immigrants reported a relatively high proportion of employment (51%) and a relatively low proportion of substance use (12%). Participants who were unhoused or chronically homeless had a high proportion of female participants (70%) than male participants (30%), likely due to significant recruitment at a women-only shelter and Housing First site. Very few participants in this group were employed (3%). Among the participants reporting substance use, a relatively high proportion (64%) were White and non-Hispanic, and a low proportion (29%) reported excellent or very good health. Participants with low income and/or uninsured had a relatively low employment rate (30%). Participants who were unhoused reported the lowest rate of COVID-19 vaccination (80%).

We ran independent 2-sample t-tests to determine differences in behavioral factors around COVID-19 testing between participants who are and are not members of each vulnerability group (Table 2). Compared to non-immigrants, immigrants were less confident in their ability to test (*P* = .001); saw less usefulness in testing with symptoms/exposure (*P* = .01); reported lower norms around COVID-19 testing (*P* = .047); and were less committed to COVID-19 testing when they experienced symptoms/exposure (*P* = .008). Participants who were unhoused or had a history of chronic homelessness reported lower norms around COVID-19 testing than participants who were stably housed (*P* = .01). Participants with a history of substance use reported a higher commitment to COVID-19 testing than participants without substance use (*P* = .02). Participants who were low income and/or uninsured were less confident in their ability to test than participants who were not low income and/or uninsured (*P* = .003). Participants who were low income and/or uninsured also had a higher perceived severity of illness from COVID-19 (*P* = .037) and lower norms around COVID-19 testing (*P* = .01).

Of the 93 total participants, 84 reported an incident (ie, exposure or symptoms) that should have prompted COVID-19 testing. Therefore, only 84 participants are included in the analysis of testing behavior. The average desired testing behavior score for these participants was 0.66, indicating that participants, on average, tested 66% of the times they should have done so, according to CDC guidelines. Overall, regardless of symptoms or exposure, participants reported taking a COVID-19 test in 425 of 1034 (41%) completed surveys.

Bivariate linear regression was conducted between the dependent variable of desired testing behavior score and each of the 7 demographic factors, 5 vulnerability characteristics, and 5 behavioral factor scores. Of these 17 variables, 4 factors (age, confidence in ability to use a COVID-19 test, usefulness of COVID-19 testing, and commitment to COVID-19 testing) were significantly associated with desired testing behavior scores (Table 3). All factors were positively associated with the primary outcome; that is, older age, greater confidence, greater perceived usefulness, and stronger commitment were associated with a higher desired testing behavior score.

We next performed a multivariable linear regression analysis with the 4 factors from the bivariate regression that were significantly associated with desired testing behavior score, as well as the vulnerability score. In the multivariable model, none of the individual factors retained a significant association with testing behavior (Table 4). The multiple regression model significantly predicted desired testing behavior scores (F[5, 77] = 6.97; *P* = .015), with an adjusted R^2^ of 0.11. These data indicate that 11% of the variance in desired testing behavior scores was explained by factors included in the model.

## 4. Discussion

We engaged communities vulnerable to COVID-19 health disparities in a year-long program of at-home COVID-19 testing to better understand COVID-19 testing behaviors among marginalized individuals and to determine what factors may be associated with desired testing behaviors. With the support of community partners, we successfully recruited and retained participants from the immigrant, unhoused, substance-use, and low-income/uninsured communities. In doing so, we laid the foundation for future studies to engage with these groups that are traditionally underrepresented in research. We report that confidence in the ability to use at-home COVID-19 tests and the commitment to testing when indicated were both relatively high across study groups, even though the perceived severity of illness from COVID-19 was fairly low. Furthermore, we found that the overall rate of taking a COVID-19 test when indicated (based on symptoms or close contact) was high across groups.

Desired COVID-19 testing behavior among participants across vulnerability groups was generally high at 66%. This rate was higher than previous research showing that overall rates of home-test use at the time were approximately 20%,^25^ and that testing rates have generally been lower among racial and ethnic minority groups^15–17^ and in lower-income areas.^18^ One explanation for this difference is that participants in this study had ready-access to, and training in the use of, COVID-19 tests, eliminating access issues as a barrier to testing. Previous studies identified many barriers to testing among underserved groups, including issues related to access, such as transportation, lack of supplies, location of testing sites, and cost of testing.^19–22^ One study distributed at-home COVID-19 tests to an underserved population through community partners and demonstrated high rates of reported testing before gatherings.^30^ Another study showed improved testing when community health workers helped reservation dwellers order home tests online.^24^ We are unaware of other home-testing studies of similar vulnerable populations. We also believe that the high rates of testing among the members of structurally vulnerable communities when provided with home tests in our study adds meaningfully to the evidence in support of providing at-home testing as a viable method to improve testing disparities.

The primary outcome variable for our study was a desired testing behavior score, which, to our knowledge, is a novel approach to defining COVID-19 testing behavior. The CDC and other health organizations do not recommend regular testing in the absence of concerns. Rather, they recommend testing for specific indications, such as symptoms concerning for COVID-19 or close contact with someone with known COVID-19.^13^ Of the studies we identified that examine testing behavior,^15,17,23,24^ all used testing rate, with no link to reasons for testing, as their outcome of interest. Our novel variable of desired testing behavior uses the instances when testing was indicated by these guidelines (denominator) and when the participant took a test when indicated (numerator). This approach (1) minimized the risk of seeing a false increase in testing rates due to participants using excessive tests simply because they are available, and (2) limited the analysis to instances when testing could have personal or public health implications. Although further study is needed, this approach to defining recommended testing could be useful in future efforts to evaluate the impact of public health programs or messaging on testing behavior.

This study was exploratory in scope and had several limitations. First, all participants received the same supply of at-home COVID-19 tests; therefore, there was no control group for direct comparison. This approach helped facilitate research engagement among a population traditionally underrepresented in biomedical research by allowing all participants to engage directly with the testing program. However, further study with a control group is needed to determine the true effect of this at-home testing program on desired testing behavior in these populations. Second, the participants recruited to this study had many overlapping vulnerability characteristics (eg, many participants who were unhoused also had low income and/or used substances), which did not allow direct group-to-group comparisons. Although this overlay reflects the overlapping vulnerabilities in underserved communities, it did not allow direct comparisons between, for example, immigrants and people who are currently unhoused. Third, although several factors showed association with desired testing behavior in bivariate analysis, no factors showed a significant independent association with desired testing in the final multivariable model. This difference indicates (1) a need to further broaden or clarify the range of factors, demographic and behavioral, that may influence testing behavior; and (2) limited statistical power. Due to a limited sample size, we did not examine desired testing behaviors in specific immigrant groups. We recognize that immigrant groups are not homogenous and may have different testing usage and behaviors. Fourth, high testing rates among participants may be partly attributable to recruitment bias, because the willingness to participate in a longitudinal testing study may correlate with more positive views on COVID-19 testing and health care seeking behavior. This possibility was supported by the high (90%) COVID-19 vaccination rate among participants.

## 5. Conclusions

This study reports high rates of desired testing behaviors among structurally vulnerable communities when barriers to accessing COVID-19 tests are removed. This study also further suggests that public health campaigns that include culturally appropriate training in COVID-19 test usage, as well as messaging about the usefulness and importance of COVID-19 testing, may increase desired testing. This study supports the approach of distributing at-home COVID-19 tests as widely as possibly to encourage desired testing behavior. Further studies examining the effect of specific approaches to public health messaging on testing behavior are needed.

## Figures and Tables

**Table 1.** Key demographics by vulnerability group.

Characteristic	Overall (N = 93)	Immigrant (n = 39)	Unhoused or history of chronic homelessness (n = 30)	Substance use (n = 28)	Low-income and/or uninsured (n = 60)
Age, y, mean (SD)	45.5 (15.0)	38.7 (12.9)	47.8 (11.8)	44.6 (12.8)	46.1 (14.1)
Gender, No. (%)					
Female	50 (54)	21 (54)	21 (70)	16 (57)	36 (60)
Male	42 (45)	18 (46)	9 (30)	12 (43)	23 (38)
Nonbinary	1 (1)	0 (0)	0 (0)	0 (0)	1 (2)
Race and Ethnicity, No. (%)					
Black and African American	34 (37)	27 (70)	9 (30)	6 (21)	24 (40)
White and non-Hispanic	45 (48)	2 (5)	15 (50)	18 (64)	26 (43)
Other	11 (12)	9 (23)	4 (13)	3 (11)	7 (12)
Employed, No. (%)	42 (45)	20 (51)	1 (3)	8 (29)	18 (30)
Education, No. (%)					
8^th^ grade or less	5 (5)	2 (5)	2 (7)	2 (7)	4 (7)
Some/all of high school	28 (30)	8 (21)	15 (50)	11 (39)	23 (38)
Some college/technical degree/vocational	22 (24)	9 (23)	10 (33)	7 (25)	18 (30)
Bachelor’s degree or higher	36 (39)	19 (49)	2 (7)	7 (25)	13 (22)
Immigrant, No. (%)	39 (42)	39 (100)	9 (30)	5 (18)	17 (28)
Low income by federal poverty guidelines, No. (%)	51 (55)	23 (59)	27 (90)	19 (68)	51 (85)
Unhoused or history of chronic homelessness, No. (%)	30 (32)	9 (23)	30 (100)	15 (54)	28 (47)
Self-reported substance use, No. (%)	28 (30)	5 (12)	15 (50)	28 (100)	20 (33)
Self-reported health excellent or very good, No. (%)	47 (51)	23 (59)	9 (30)	8 (29)	28 (47)
Health insurance, No. (%)					
Private	27 (29)	12 (31)	0 (0)	6 (21)	4 (7)
Public (Medicare, Medicaid, Tricare)	45 (48)	13 (33)	23 (77)	20 (71)	36 (60)
Uninsured	17 (18)	12 (31)	6 (20)	1 (4)	17 (28)
Received COVID-19 vaccine, No. (%)	84 (90)	36 (92)	24 (80)	25 (89)	52 (87)

**Table 2.** COVID-19 testing behavioral factors on 5-point likert scales by vulnerability group*.

	Confidence in ability to use COVID-19 tests		Perceived severity of illness from COVID-19		Usefulness of COVID-19 testing		Norms around COVID-19 testing		Commitment to COVID-19 testing	
	Mean (SD)	*P* value	Mean (SD)	*P* value	Mean (SD)	*P* value	Mean (SD)	*P* value	Mean (SD)	*P* value
All participants (n = 92)	4.16 (0.78)		2.15 (0.89)		4.12 (1.02)		2.78 (0.92)		4.05 (0.83)	
Immigrants (n = 39)	**3.80 (0.76)**	**.0001**	2.09 (0.83)	.60	**3.80 (1.07)**	**.01**	**2.56 (0.89)**	**.047**	**3.78 (0.85)**	**.0077**
Unhoused or history of chronic homelessness (n = 29)	4.02 (0.73)	.23	2.38 (0.97)	.08	4.02 (0.97)	.54	**2.43 (0.87)**	**.0096**	4.07 (0.84)	.65
Substance use (n = 39)	4.30 (0.74)	.25	2.31 (0.84)	.26	4.39 (0.82)	.10	2.86 (0.89)	.60	**4.35 (0.66)**	**.0203**
Low-income/Uninsured (n = 52)	**3.95 (0.80)**	**.0003**	**2.29 (0.91)**	**.037**	4.08 (0.97)	.61	**2.60 (0.89)**	**.0097**	3.98 (0.81)	.29

* Differences in testing analyzed using independent-sample t-tests comparing group vs non-group (eg, immigrants vs non-immigrants).

Bold indicates statistical significance (*P* < .05).

**Table 3.** Bivariate Linear Regression to Assess Association Between Demographic, Vulnerability, and Behavioral Factors and Desired COVID-19 Testing Behavior Scores.

Dependent variable	Value	Beta coefficient	*P* value^†^
Desired testing behavior score,* mean (SD)	0.66 (0.29)	NA	NA
**Demographics**			
Age, y, mean (SD)^‡^	44.81 (14.41)	0.005	**.035**
Gender, No. (%)			
Female	48 (57)	−0.044	.503
Male	35 (42)	Reference	Reference
Nonbinary	1 (1)	−0.186	.535
Education, No. (%)			
8^th^ grade or less	5 (6)	Reference	Reference
Some or all of high school	26 (31)	−0.052	.709
Some college/technical degree/vocational degree	18 (21)	−0.092	.524
Bachelor’s degree	23 (27)	−0.251	.079
Advanced degree	11 (13)	−0.015	.924
Race, No. (%)			
White and non-Hispanic	41 (49)	Reference	Reference
Black and African American	29 (35)	−0.067	.344
Other	14 (17)	−0.153	.093
Reported health, mean (SD)^§^	2.60 (1.04)	−0.029	.352
Employed, No. (%)	37 (44)	0.002	.280
Received COVID-19 vaccine	75 (89)	−0.003	.259
**Social and economic vulnerabilities**			
Immigrant, No. (%)	35 (42)	−0.092	.157
Unhoused or history of chronic homelessness, No. (%)	29 (35)	0.093	.166
Substance use, No. (%)	28 (30)	0.005	.947
Low-income and/or uninsured, No. (%)	54 (64)	0.065	.329
Vulnerability score, mean (SD)	1.7 (1.07)	0.013	.664
**Behavioral factors** ^§^			
Confidence in ability to use COVID-19 tests, mean (SD)	4.18 (0.79)	0.095	**.017**
Perceived severity of illness from COVID-19, mean (SD)	2.18 (0.92)	0.035	.114
Perceived norms around COVID-19 testing, mean (SD)	2.80 (0.94)	−0.019	.586
Commitment to COVID-19 testing, mean (SD)	4.06 (0.84)	0.112	.**003**
Usefulness of taking COVID test with symptoms/contact, mean (SD)	4.13 (1.06)	0.085	**.006**

Abbreviation: NA, not applicable.

* Proportion of instances a participant took a COVID-19 test when they should have.

† Bold indicates statistical significance (*P* < .05).

‡ 1-year increment.

§ Likert scale from 1 to 5.

**Table 4.** Multivariable linear regression of factors associated with desired COVID-19 testing behavior scores (n = 83)*.

Variable	Beta (95%CI)	SE	*P* value
Age	0.004 (−0.0002 to 0.008)	0.002	.062
Usefulness of COVID-19 testing	0.032 (−0.068 to 0.131)	0.050	.527
Confidence in ability to use COVID-19 tests	0.068 (−0.032 to 0.167)	0.059	.179
Commitment to COVID-19 testing	0.036 (−0.093 to 0.165)	0.065	.582
Vulnerability score	0.038 (−0.024 to 0.101)	0.031	.229

* Adjusted R^2^ = 0.11.

## Appendices

### A. Methods details

#### A.1. Community engaged approach

We utilized a community engaged approach throughout the study design and implementation, primarily through collaboration with community partners and a Community & Scientific Advisory Board (CSAB). Our three community partner organizations were: Preble Street (a homeless services and food security agency), PCFC (a volunteer clinic for low-income, uninsured people), and Greater Portland Health (GPH, a federally-qualified health center that primarily serves the immigrant community). Community partners informed recruitment and survey approaches, provided insights into the importance of offering a variety of survey formats (email, text, WhatsApp, in-person), and facilitated recruitment as trusted entities within the participant communities. CSAB members included directors and leaders from government, non-profit, and healthcare sectors who serve the populations of interest. The CSAB met regularly throughout the study and provided feedback on recruitment and survey approaches, including adjustments to compensation amounts and frequency of surveys to enhance participant retention.

#### A.2. Recruitment

Recruitment occurred in-person at our community partner organizations, as well as at the STD Clinic and Needle Exchange, and at Housing First sites run by Preble Street (permanent apartments for people with a history of chronic homelessness). Additional recruitment efforts included advertising through community partner staff and CSAB members; posting flyers and posters at community partner sites, clinic waiting rooms, and public locations such as libraries and ethnic grocery stores; meeting with and providing flyers to relevant community organizations; posting on the MaineHealth Facebook page; advertising in a print newspaper serving the African immigrant community; utilizing the social networks of study staff from the immigrant community; and advertising the study to people utilizing COVID-19 testing walk-up clinics conducted in a related study. All recruitment materials were translated into seven languages (Somali, Arabic, French, Kinyarwanda, Lingala, Portuguese and Spanish) representing the top languages spoken by patients of GPH. Despite recruitment efforts in seven languages, enrolled participants spoke one of three languages (English, French, and Portuguese). Research staff fluent in the participant’s primary spoken language completed the enrollment process.

#### A.3. Measures: Variable definitions

Participants were included in vulnerability groupings based on self-reported measures from the enrollment survey. A participant was categorized as an immigrant if they reported a country of birth outside the United States. A participant was categorized as unhoused or having a history of chronic homelessness if they reported homelessness or were recruited from a shelter or Housing First location. A participant was categorized as low-income if the participant’s reported household income from 2019 was below the 2019 federal poverty limit^1^ for their given household size, and as uninsured if the participant reported they did not have health insurance. A participant was categorized as having substance use based on a positive response to the NIDA-Modified ASSIST survey (weekly or more often binge-drinking, illicit drug use, or prescription drug use for non-prescribed indications) or if they self-identified as having an alcohol use or substance use disorder on the health screening.^2^

Behavioral factor scores were based on five-point Likert scale questions. Participants responded to behavioral questions at enrollment and every eight weeks for a total of seven responses per question; these were averaged to generate a single score on a scale of one to five for each participant for each question. Qualitative findings from an earlier part of this study, as well as previously published research on perceived barriers and facilitators of COVID-19 testing,^3–5^ helped to identify types of behavioral factors linked to testing in these communities and also informed development of survey items.

#### A.4. Common data elements (CDE)

The RADx-UP Common Data Elements (CDEs) were derived from sources including the National Institutes of Health (NIH) CDE Repository, Disaster Research Response (DR2) guidelines, and the PHENotypes and eXposures (PhenX) Toolkit. The codebooks and sample forms are publicly available onRADx-UP.org, the NIHDR2website, and the NIHNationalLibraryofMedicineCDEBrowser.^6^

The datasets for this manuscript are not yet publicly available because RADx data have not yet been made available to the public for review and analysis. Requests to access the datasets should be directed to the NIHRADxDataHub.

### B. Tables

**Table A1.** Demographic, health, and vulnerability characteristics of study participants.

Demographics	N (93)	%
Age (mean 45.5 years)		
18–24 years	7	8%
25–39 years	33	35%
40–59 years	34	37%
60 years and older	19	20%
Gender		
male	42	45%
female	50	54%
Non-binary	1	1%
Race/Ethnicity		
Black/African-American	34	37%
White, non-Hispanic	45	48%
Hispanic	5	5%
Asian	3	3%
Native American	1	1%
Other	2	2%
Employed currently	42	45%
Household Income		
Less than $15,000	36	39%
$15,000–24,999	17	18%
$25,000–49,999	10	11%
$50,000 and higher	16	17%
Education Level		
8^th^ grade or less	5	5%
Some/all high school	28	30%
Some college/technical degree/vocational	22	24%
Bachelor’s degree or higher	36	39%
**Health characteristics**		
Self-reported health		
Excellent	16	17%
Very good	31	33%
Good	28	30%
Fair	15	16%
Poor	3	3%
Received COVID-19 vaccine	84	90%
**Vulnerability characteristics**		
Immigrant	39	42%
Africa	29	32%
Asia	3	3%
Latin America	4	4%
Middle East	3	3%
Low income by federal poverty guidelines	51	55%
Health Insurance		
Private	27	29%
Public (Medicare, Medicaid, Tricare)	45	48%
Uninsured	17	18%
Unhoused or history of chronic homelessness	30	32%
Self-reported substance use	28	30%

**Table A2.** Behavioral factor questions.

How confident are you in your ability to properly conduct an at-home COVID-19 test?	Not confident (1) – Very confident (5)
Imagine you test positive for COVID-19. How severely do you think your health would be impacted?	Not severe (1) – Very severe (5)
How *useful* do you think it is to take a COVID-19 test when you are experiencing symptoms or had a close contact?	Not at all useful (1) – Very useful (5)
Among the people you know, how many take a COVID-19 test when they are experiencing symptoms or had a close contact?	(Almost) none of them (1) – (Almost) all of them (5)
How committed are you to taking a COVID-19 test when you are experiencing symptoms or had a close contact?	Not at all committed (1) – Very committed (5)
